# Impact of low-dose CT screening on smoking cessation among high-risk participants in the UK Lung Cancer Screening Trial

**DOI:** 10.1136/thoraxjnl-2016-209690

**Published:** 2017-07-14

**Authors:** Kate Brain, Ben Carter, Kate J Lifford, Olivia Burke, Anand Devaraj, David R Baldwin, Stephen Duffy, John K Field

**Affiliations:** 1 Division of Population Medicine, Cardiff University School of Medicine, Cardiff, UK; 2 Department of Biostatistics and Health Informatics, Institute of Psychiatry, Psychology and Neuroscience, King's College London, London, UK; 3 Royal Brompton and Harefield NHS Trust, London, UK; 4 Department of Respiratory Medicine, Nottingham University Hospitals, Nottingham, UK; 5 Queen Mary University of London, London, UK; 6 Roy Castle Lung Cancer Research Programme, Department of Molecular and Clinical Cancer Medicine, University of Liverpool, Liverpool, UK

**Keywords:** Lung Cancer, Smoking cessation

## Abstract

**Background:**

Smoking cessation was examined among high-risk participants in the UK Lung Cancer Screening (UKLS) Pilot Trial of low-dose CT screening.

**Methods:**

High-risk individuals aged 50–75 years who completed baseline questionnaires were randomised to CT screening (intervention) or usual care (no screening control). Smoking habit was determined at baseline using self-report. Smokers were asked whether they had quit smoking since joining UKLS at T_1_ (2 weeks after baseline scan results or control assignment) and T_2_ (up to 2 years after recruitment). Intention-to-treat (ITT) regression analyses were undertaken, adjusting for baseline lung cancer distress, trial site and sociodemographic variables.

**Results:**

Of a total 4055 individuals randomised to CT screening or control, 1546 were baseline smokers (759 intervention, 787 control). Smoking cessation rates were 8% (control n=36/479) versus 14% (intervention n=75/527) at T_1_ and 21% (control n=79/377) versus 24% (intervention n=115/488) at T_2_. ITT analyses indicated that the odds of quitting among screened participants were significantly higher at T_1_ (adjusted OR (aOR) 2.38, 95% CI 1.56 to 3.64, p<0.001) and T_2_ (aOR 1.60, 95% CI 1.17 to 2.18, p=0.003) compared with control. Intervention participants who needed additional clinical investigation were more likely to quit in the longer term compared with the control group (aOR 2.29, 95% CI 1.62 to 3.22, p=0.007) and those receiving a negative result (aOR 2.43, 95% CI 1.54 to 3.84, p<0.001).

**Conclusions:**

CT lung cancer screening for high-risk participants presents a teachable moment for smoking cessation, especially among those who receive a positive scan result. Further behavioural research is needed to evaluate optimal strategies for integrating smoking cessation intervention with stratified lung cancer screening.

**Trial registration number:**

Results, ISRCTN 78513845

**Key messages**

**What is the key question?**

- What is the effect on smoking cessation of taking part in the UK randomised pilot trial of low-dose CT lung screening?

**What is the bottom line?**

- CT lung cancer screening does not appear to falsely reassure smokers or reduce their motivation to stop smoking.

**Why read on?**

- For clinicians and policy makers who are considering implementation of stratified (ie, high-risk) lung cancer screening, this study adds to evidence suggesting that integrating CT screening with evidence-based smoking cessation interventions could prompt quitting in motivated high-risk smokers.

## Introduction

Smoking is the leading cause of preventable morbidity and premature mortality worldwide.^1^ In the UK, an estimated 86% of lung cancer cases are attributable to smoking.^2^ The prevalence of cigarette smoking in the UK remained relatively stable between 2006 and 2014 at approximately 10 million adults (∼20%)^3^ and although these rates are much lower than those of the 1970s, this declining trend has begun to plateau.^4^ The association between cigarette smoking and socioeconomic group is well established, with higher smoking rates among people living in more deprived areas.^5^

Trials have been undertaken to ascertain the effectiveness of low-dose CT screening for the earlier detection of lung cancer in high-risk groups, including smokers.^6–9^ The impact of CT lung screening on smoking cessation and abstinence has been examined in response to concerns that taking part in lung screening may offer a ‘licence to smoke’, especially for smokers who receive favourable screening results.^10^ Evidence from controlled trials, however, suggests that participating in lung screening significantly increases smoking cessation rates overall compared with the general population, and that receiving a positive CT screening result may provide an additional cue to action in prompting cessation. The Danish Lung Cancer Screening Trial (DLCST) reported smoking cessation rates of almost 12% in both trial arms at 1 year follow-up, compared with the Danish population rate of 4%.^11^ Quit rate was significantly higher in smokers who had a positive CT result that required repeat scans.^11^ In the Dutch-Belgian NELSON Trial, the overall quit rate at 2 years follow-up was 16.6% compared with a background population rate of 3–7%. Although a lower prolonged abstinence rate was observed in the screened arm (14.5%) versus control (19.1%), this effect disappeared following intention-to-treat (ITT) analysis, suggesting an overall positive effect of trial participation.^12^ A non-significant trend towards increased smoking cessation was seen in those with multiple indeterminate screening results.^13^ The US National Lung Screening Trial (NLST)^14^ found that compared with normal lung screening results, receiving any screen-detected abnormality significantly reduced the probability of continued smoking.

In addition to the moderating effect of lung screening results, demographic predictors of increased likelihood of smoking cessation have been observed in previous lung cancer screening studies. These have included older age,^11^ ^14^ ^15^ higher socioeconomic group,^14^ higher education,^12^ being married^14^ and lower nicotine dependency.^11^ ^14^ In addition, participants with higher levels of concern about lung cancer and greater perceived benefits of stopping smoking,^10^ and those who intend to stop smoking,^11^ ^12^ are more likely to quit in the context of lung cancer screening.

The UK Lung Cancer Screening (UKLS) Pilot Trial is the first to assess the feasibility, cost-effectiveness and psychosocial impact of lung cancer screening using a single low-dose CT screen versus no screening in a UK high-risk population.^9^ ^16^ The current study builds upon previous UKLS reports by examining the behavioural effects of trial participation and modifying variables on smoking cessation at short-term and long-term follow-up. It was expected that intervention arm participants would be more likely to report quitting compared with control arm participants, and that predictors of smoking cessation would include positive CT results (in screened participants), higher socioeconomic group and higher baseline distress/concern about lung cancer.

## Methods

### Participants and procedures

A random sample of 247 354 individuals aged 50–75 years residing in six recruitment areas in the UK (Liverpool, Knowsley and Sefton; Cambridgeshire, Peterborough and Bedfordshire) was sent trial information packs that included a self-report questionnaire regarding lung cancer risk factors. From the questionnaire responders, 8729 patients were identified as high risk of lung cancer (≥5% over 5 years) using the Liverpool Lung Project (LLP_v2_) risk prediction model which includes age, sex, family history of lung cancer, smoking duration, personal history of other cancers and non-malignant respiratory diseases and exposure to asbestos.^9^ Characteristics of trial non-participants are reported elsewhere.^17^ ^18^

Following completion of a second questionnaire to identify trial eligibility, those meeting the criteria were invited to attend their local recruitment centre in Liverpool or Cambridge (trial sites). High-risk individuals who gave informed written consent were randomised on a 1:1 ratio to the intervention (screening) or control arms. Randomisation used unique random personal ID codes and computer-generated sequencing for allocation concealment.^9^ Participants who self-reported smoking in the first questionnaire were eligible for inclusion in the current analyses. Participants in both trial arms were offered standard smoking cessation advice leaflets and given a list of local National Health Service Stop Smoking services prerandomisation.

Participants completed a touchscreen questionnaire that included baseline psychosocial measures (T_0_). A second psychosocial questionnaire (T_1_) was sent approximately 2 weeks after receiving either a letter of assignment to the control group or a baseline CT scan result letter (intervention arm). T_2_ psychosocial questionnaires were sent in a single mailshot during January 2014.

### Measures

*Smoking status* was calculated at T_0_ based on self-report data within the first UKLS questionnaire. Participants were categorised into current smokers, ex-smokers and never smokers.

*Smoking cessation* was assessed using self-report at T_1_ and T_2_. Participants were asked whether they had quit smoking since joining UKLS, with response options ‘yes’, ‘no’, ‘no but I intend to quit smoking within the next 6 months’ and ‘not applicable’ (ie, not a smoker at baseline). Participants who responded ‘no’ or ‘no, but intend to quit’ were categorised as non-quitters. Those who responded ‘not applicable’ or who returned the questionnaire but missed out the smoking cessation question were categorised as non-completers.

*Lung cancer distress* was measured using six items adapted from Lerman *et al*^19^ and Watson *et al*^20^ to assess the frequency of lung cancer-related thoughts and their impact on mood and daily functioning. Total score range was 6–24, with a score above 12.5 corresponding to a clinically significant threshold score on the General Health Questionnaire-28.^21^

*Demographic variables:* age and gender were obtained from medical records. Socioeconomic group was measured using Index of Multiple Deprivation ranks calculated from postcodes and categorised into standard deprivation quintiles (quintile 1=most deprived, quintile 5=least deprived). Marital group and experience of lung cancer (self and/or close others) were included in the T_0_ questionnaire

### Screening results

Baseline CT scan results in the intervention arm included negative (normal) results, those requiring a repeat scan in 3 or 12 months, those requiring referral to the multidisciplinary team due to a major lung abnormality and significant incidental findings (such as aortic aneurisms and pneumonia but with no findings suspicious for lung cancer).

### Statistical analysis

Analyses were conducted using Stata V.14. Baseline comparisons were undertaken to compare the characteristics of smokers who did and did not complete follow-up questionnaires. Participants who did not answer the smoking cessation question at T_1_ or T_2_ were imputed as smokers and included in the primary analysis of intervention effect using the ITT population, in accordance with the Russell Standard for reporting smoking cessation trials.^22^ Complete case sensitivity analyses were also conducted, using univariable logistic regression models fitted to the smoking cessation outcome data at T_1_ and T_2_ independently with an inverse probability weighting.^23^ Additionally, as a secondary analysis to adjust for confounders, multivariable logistic regression models were fitted to evaluate the impact of trial allocation on smoking cessation at T_1_ and T_2_ adjusting for T_0_ lung cancer distress, sociodemographic factors (gender, age group, marital group, deprivation quintile, experience of lung cancer) and trial site.^16^ ORs and adjusted ORs (aORs) with 95% CIs and p values are presented. Due to multiple testing, p<0.01 was used to denote statistical significance.

To investigate the effect of the baseline scan result on smoking cessation, we summarised intervention arm participants into those who had a scan leading to additional clinical investigation (including repeat scan, major abnormality and incidental findings) and those receiving a negative result (ie, not requiring further investigation). Participants randomised to the control group were used as the reference category in order to reflect current practice. The impact of additional clinical investigation on smoking cessation at T_1_ and T_2_ was analysed using univariable and multivariable logistic regressions in the imputed and complete case populations. Lung cancer distress, sociodemographic factors and trial site were included in multivariable analysis.

Further subgroup analyses were carried out within the intervention arm only, involving additional univariable regression analyses to examine T_1_ and T_2_ smoking cessation in those receiving additional clinical investigation compared with a negative result as the reference category. We carried out this analysis to reflect a potential national policy where participants receive routine lung screening.

## Results

### Trial participation

**Figure 1:**
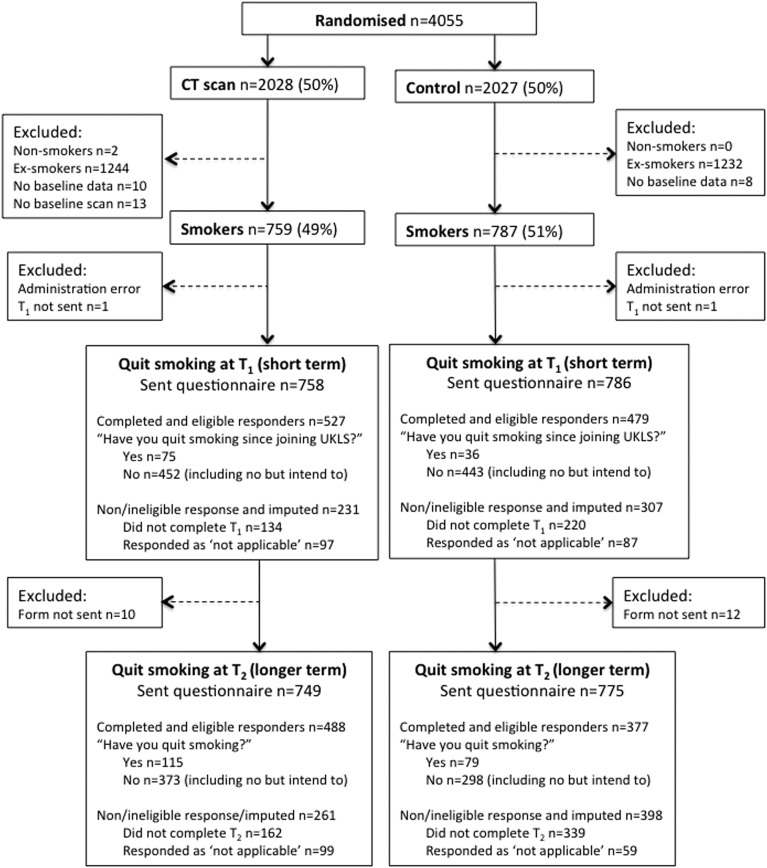
Trial CONSORT diagram. UKLS, UK Lung Cancer Screening.

In total, 4061 individuals (5% of 75 958 responders to the risk questionnaire; 47% of all high-risk positive responders) attended the recruitment clinic and were consented.^24^ As shown in figure 1, 4055 trial participants were randomised (n=2028 CT intervention, n=2027 control). T_1_ completion rates were n=527/758 (70%) for the intervention arm and n=479/786 (61%) for the control arm, giving a total T_1_ sample of n=1006. The non-completion rate at T_1_ was n=538, of whom 231 (43%) were intervention and 307 (57%) were control participants. T_2_ completion rates were n=488/749 (65%) for the screening arm and n=377/775 (49%) for the control arm (total T_2_ n=865). Of the 659 T_2_ non-completers, 261 (40%) were from the intervention arm and 398 (60%) were from the control arm.

### Factors associated with non-completion

Baseline smokers in the control arm and those with experience of lung cancer were significantly less likely than those in the intervention arm to complete T_1_ questionnaires (see online supplementary table SI). Trial site, age, gender, marital group, deprivation and T_0_ lung cancer distress were not statistically significantly associated with T_1_ completion.

10.1136/thoraxjnl-2016-209690.supp1 Supplementary table I

T_2_ questionnaire completion was significantly lower among baseline smokers in the control arm and those recruited at the Liverpool site in the most deprived quintile and with experience of lung cancer. Age, marital group, gender and baseline distress were not statistically significantly associated with T_2_ completion (see online supplementary table SII).

10.1136/thoraxjnl-2016-209690.supp2 Supplementary table II

### Effect of trial allocation on T_1_ and T_2_ smoking cessation

As shown in figure 1, the overall T_1_ trial quit rate was 111/1006 (11%). In the screening arm, 75 (14%) individuals quit smoking at T_1_ and 452 (86%) continued to smoke. Thirty-six individuals (8%) in the control arm had quit smoking at T_1_ compared with 443 (92%) who had not quit. At T_2_, the overall quit rate was 194/865 (22%), with 115 screened individuals (24%) and 79 (21%) individuals in the control arm having quit smoking.

Primary ITT and sensitivity analyses are summarised in table 1. T_1_ smoking cessation was statistically significantly higher in screened individuals compared with control (p<0.001) and remained statistically significant (p=0.001) after adjusting for T_0_ distress and all other covariates. Sensitivity analysis confirmed that T_1_ smoking cessation was statistically significantly higher in the intervention group (p<0.001). Effects of covariates on T_1_ smoking cessation were not statistically significant in ITT analyses (see online supplementary table SIII): trial site (p=0.14); age group compared with those under 65 years (66–70 years p=0.50, over 70 years p=0.81); gender (p=0.87); marital group (p=0.38); deprivation (most vs least deprived p=0.50) and lung cancer experience (p=0.45). There was limited evidence that participants with higher baseline lung cancer distress were more likely to quit smoking at T_1_ (p=0.03). Similarly, covariates were non-significant in crude and adjusted complete case analyses (see online supplementary table SIV).

10.1136/thoraxjnl-2016-209690.supp3 Supplementary table III

10.1136/thoraxjnl-2016-209690.supp4 Supplementary table IV

**Table 1:** Effect of trial allocation on T_1_ and T_2_ smoking cessation

*Quit smoking at T_1_ (n=1544)*				
Primary analysis*	Yes (n=111)	No (n=1433)	Univariable OR (95% CI)	Multivariable OR† (95% CI)
Intervention, n (%)	75 (68%)	683 (48%)	2.29 (1.52 to 3.45)	2.38 (1.56 to 3.64)
Control, n (%)	36 (32%)	750 (52%)		
Sensitivity analysis‡	Yes (n=111)	No (n=895)	Univariable OR (95% CI)	Multivariable OR† (95% CI)
Intervention, n (%)	75 (68%)	452 (50%)	2.04 (1.34 to 3.10)	2.09 (1.36 to 3.23)
Control, n (%)	36 (32%)	443 (50%)		
***Quit smoking at T_2_ (n=1524)***				
Primary analysis*	Yes (n=194)	No (n=1330)	Univariable OR (95% CI)	Multivariable OR† (95% CI)
Intervention, n (%)	115 (59%)	634 (48%)	1.60 (1.18 to 2.17)	1.60 (1.17 to 2.18)
Control, n (%)	79 (41%)	696 (52%)		
Sensitivity analysis‡	Yes (n=194)	No (n=671)	Univariable OR (95% CI)	Multivariable OR† (95% CI)
Intervention, n (%)	115 (59%)	373 (55%)	1.16 (0.84 to 1.61)	1.16 (0.65 to 1.33)
Control, n (%)	79 (41%)	298 (45%)		

*Intention-to-treat (ITT) analyses with the imputed population.

†Adjusted for T_0_ cancer distress, recruitment site, gender, age, marital group, deprivation and experience of lung cancer.

‡Complete case analyses.

At T_2_, there was a statistically significant difference between trial arms in quitting smoking in the crude ITT analysis (p=0.003) and after adjusting for covariates (p=0.003). In sensitivity analyses, the difference in cessation rate between intervention and control groups at T_2_ was not statistically significant (p=0.36); therefore, these findings should be interpreted with caution (see table 1). The effects of other variables on T_2_ smoking cessation rate were not statistically significant in ITT analyses (see online supplementary table SV): trial site (p=0.28); age (66–70 years p=0.49; over 70 years p=0.18); gender (p=1.18); marital group (p=0.07); deprivation (most vs least deprived p=0.24); lung cancer experience (p=0.60) and baseline distress (p=0.08). The effects of covariates were not statistically significant in complete case analyses at T_2_ (see online supplementary table SVI).

10.1136/thoraxjnl-2016-209690.supp5 Supplementary table V

10.1136/thoraxjnl-2016-209690.supp6 Supplementary table VI

### Impact of additional clinical investigation on smoking cessation

T_1_ smoking cessation was reported by 16% (48/299) of participants who had additional clinical investigation following the baseline scan result and 11% (26/227) who received a negative result. These were both compared with 8% (36/479) who reported T_1_ smoking cessation in the control group. As shown in table 2, the impact of needing additional clinical investigation on T_1_ smoking cessation was statistically significant in univariable (p<0.001) and multivariable (p<0.001) analyses using the imputed population. The effect of receiving a negative result on T_1_ smoking cessation was not significant in univariable (p=0.09) and multivariable (p=0.09) analyses. Similar findings were observed in sensitivity analyses for both T_1_ comparisons.

**Table 2:** Impact of baseline scan result on T_1_ and T_2_ smoking cessation

Baseline scan result		Quit smoking	Total	Univariable OR (95% CI)	Multivariable OR* (95% CI)
Primary analysis†					
T_1_	Control group	36	786	–Reference–	–Reference–
	Negative result‡	26	340	1.73 (1.02 to 2.91)	1.78 (1.04 to 3.05)
	Additional investigation‡	48	416	2.72 (1.73 to 4.26)	2.85 (1.79 to 4.53)
T_2_	Control group	79	775	–Reference–	–Reference–
	Negative result‡	32	338	0.92 (0.60 to 1.42)	0.90 (0.58 to 1.40)
	Additional investigation‡	83	409	2.24 (1.60 to 3.14)	2.29 (1.62 to 3.22)
Sensitivity analysis§					
T_1_	Control group	36	479	–Reference–	–Reference–
	Negative result	26	227	1.59 (0.94 to 2.71)	1.61 (0.93 to 2.77)
	Additional investigation‡	48	299	2.35 (1.49 to 3.72)	2.46 (1.53 to 3.96)
T_2_	Control group	79	377	–Reference–	–Reference–
	Negative result	32	212	0.67 (0.43 to 1.05)	0.65 (0.41 to 1.04)
	Additional investigation‡	83	275	1.63 (1.14 to 2.33)	1.66 (1.15 to 2.39)

*Adjusted for T_0_ cancer distress, recruitment site, gender, age, marital group, deprivation and experience of lung cancer.

†Intention-to-treat (ITT) analyses with the imputed population.

‡One participant removed due to protocol deviation.

§Complete case analyses.

At T_2_, 30% (83/275) of participants who received additional clinical investigation following the baseline scan result and 15% (32/212) who had negative results reported cessation. These were compared with 21% (79/377) in the control group who reported quitting at T_2_. There was a statistically significant effect of additional clinical investigation on T_2_ smoking cessation in univariable (p=0.007) and multivariable (p=0.007) ITT analyses. The effect of a negative result on T_2_ smoking cessation was not significant in univariable (p=0.08) and multivariable (p=0.07) analyses; however, caution is needed when interpreting these findings. Similar results were observed in complete case analyses (see table 2).

### Subgroup analyses in the intervention group only

At T_1_, there was no statistically significant effect of needing additional clinical investigation on smoking cessation, compared with receiving a negative result (aOR 1.48, 95% CI 0.89 to 2.47, p=0.09). At T_2_, there was a clear effect on smoking cessation of additional clinical investigation compared with a negative result (aOR 2.43, 95% CI 1.54 to 3.84, p<0.001). Similar results were found in complete case analyses (results not shown).

## Discussion

Tobacco control is the major primary prevention option for lung cancer. The present study is the first to report the behavioural impact of CT screening in a UK high-risk population and confirms the findings of previous trials that lung cancer screening does not falsely reassure smokers or reduce their motivation to stop smoking. The net UKLS Trial cessation rate was 11% in the short term and 22% at up to 2 years follow-up—both higher than the background cessation rate of 4% in the general UK population. For participants who underwent CT screening, the short-term quit rate of 14% was similar to that of the Dutch-Belgian NELSON Trial^12^ and higher than that of the DLCST.^11^ Participating in the UKLS appeared to prompt smoking cessation overall, with a differential and positive effect of lung screening at short-term and longer-term follow-up. While a degree of caution is needed due to imputation of missing responders as smokers,^22^ the present findings indicate that smoking cessation was higher in the intervention arm and that engaging in CT lung cancer screening increased the likelihood of stopping smoking in the longer term. Despite concerns about a negative lung screen offering a ‘licence to smoke’,^10^ there was no evidence that UKLS screening participants who received an all-clear CT result were less likely to quit. Analysis indicated that a positive CT scan result prompted smoking cessation in the longer term compared with participants who were not screened and participants who received a negative scan, suggesting that a positive lung screening result may provide an additional stimulus for quitting over and above that of screening participation. This mirrors the findings of other controlled trials, including the DLCST^11^ and US NLST,^14^ where smokers with identified abnormalities were more likely to stop smoking than those with normal results.

The current evidence suggests that an integrated package of CT lung screening and smoking cessation support has the potential to expedite quitting in smokers who are motivated and receptive. The voluntary nature of the trial meant that smokers who took part were self-selected and may already have been contemplating quitting.^25^ It is difficult, therefore, to directly attribute smoking cessation to UKLS participation, although Ostroff *et al*^10^ reported that smokers who quit after CT lung screening ascribed their decision to screening participation. In the current trial, we observed a marginal trend towards higher baseline distress in those who reported quitting in the short term, which suggests that experiencing a degree of concern about lung cancer may be necessary to galvanise smoking cessation. However, we did not directly assess baseline quit intentions, and future evaluations of CT lung screening would therefore benefit from examining the influence of both mood-related and smoking-related cognitions on behavioural outcomes.

The limitations of sample size and study design are acknowledged. The UKLS Trial was not specifically designed to test the effects of lung screening combined with smoking cessation support; hence, the types of stop-smoking interventions accepted by participants were not recorded, nor were comparative data available on cessation rates in the Liverpool and Papworth regions during the life of the trial. It was not possible to ascertain the moderating role of nicotine dependence or biochemically validate self-reported smoking behaviour; therefore, the current findings should be interpreted cautiously due to the sole use of self-reported cessation. Nevertheless, the present study adds to growing evidence that integrating CT lung screening with evidence-based smoking cessation interventions could prompt quitting in motivated high-risk smokers. While our sample was not sufficiently large to examine continued smoking abstinence in those who reported quitting at short-term follow-up, the NELSON Trial indicated that combining low-dose CT screening with smoking cessation advice led to sustained abstinence.^12^ Most smokers enrolling in CT lung screening studies are motivated to quit; therefore, it will be critical to evaluate actual quit rates prompted by screening and whether they are maintained over time in the context of a lung screening health service. We found that longer-term study retention was less likely in smokers who were from socioeconomically deprived areas and who had experience of lung cancer. Evidence from other studies suggests that these high-risk groups may be deterred from lung screening due to fearful and fatalistic beliefs about lung cancer outcomes,^26–28^ stigma and scepticism.^29–31^ The consistent association between smoking, deprivation and lower screening uptake is a problem for public health that must be addressed in future lung screening.

Implementation of a UK national lung cancer screening programme for high-risk groups offers opportunities for smoking cessation at multiple points in the screening process, from the initial screening invitation to CT scanning and disclosure of results.^10^ Smoking cessation counselling combined with pharmacotherapy is effective^32–34^ and could be successfully implemented in the lung screening setting.^35^ However, further behavioural research is needed to identify ways of engaging harder to reach smokers and to robustly test the optimal type and timing of strategies for delivering stop-smoking support to high-risk participants. Successful integration of evidence-based strategies for smoking cessation with stratified lung cancer screening could be a prudent use of limited healthcare resources, translating into major health benefits for all smoking-related diseases.
