# Supplementary material for: Impact of low-dose CT screening on smoking cessation among high-risk participants in the UK Lung Cancer Screening Trial
Source: Thorax. 2017 Jul 14;72(10):912–8. doi: 10.1136/thoraxjnl-2016-209690 (PMC5738533; doi:10.1136/thoraxjnl-2016-209690)
Supplement: Supplementary table I [file thoraxjnl-2016-209690supp001.pdf]

Supplementary Table I. Baseline comparison of smokers who did/did not complete T_1 questionnaires

		T_1 completers (n=1006) n (%) or mean (SD)	T_1 non-completers (n=538)[‡] n (%) or mean (SD)	P value
**Trial allocation**	Intervention	527 (52%)	231 (43%)	<0.001
	Control	479 (48%)	307 (57%)	
**Site**	Liverpool	540 (54%)	315 (59%)	0.08
	Cambridge	466 (46%)	223 (41%)	
**Age**	Up to 65 years	408 (66%)	206 (34%)	0.02
	66 – 70 years	444 (67%)	220 (33%)	
	Over 70 years	154 (58%)	112 (42%)	
**Gender**	Male	701 (70%)	381 (71%)	0.69
	Female	305 (30%)	157 (29%)	
**Marital group**	Married/cohabiting	668 (67%)	352 (65%)	0.68
	Not married/cohabiting	335 (33%)	186 (35%)	
**IMD**	Quintile 1 (most deprived)	346 (34%)	183 (34%)	0.07
	Quintile 2	109 (11%)	81 (15%)	
	Quintile 3	160 (16%)	93 (17%)	
	Quintile 4	162 (16%)	82 (15%)	
	Quintile 5 (least deprived)	229 (23%)	99 (18%)	
**Lung cancer experience**	No	600 (60%)	273 (51%)	<0.001
	Yes	403 (40%)	264 (49%)	
**Cancer distress (T_0)**[+]		2.24 (0.29) *9.77 (2.87)*	2.23 (0.32) *9.81 (3.34)*	0.60

Note: percentages were calculated based on available data.

[‡] Non-completers included non-responders and ineligible responders.

[+] Log_n scores in normal text, original scores in italics (analyses performed using log_n scores).
