# Supplementary material for: Impact of low-dose CT screening on smoking cessation among high-risk participants in the UK Lung Cancer Screening Trial
Source: Thorax. 2017 Jul 14;72(10):912–8. doi: 10.1136/thoraxjnl-2016-209690 (PMC5738533; doi:10.1136/thoraxjnl-2016-209690)
Supplement: Supplementary table II [file thoraxjnl-2016-209690supp002.pdf]

Supplementary Table II. Baseline comparison of smokers who did/did not complete T_2 questionnaires

		T_2 completers (n=865) n (%) or mean (SD)	T_2 non-completers (n=661)‡ n (%) or mean (SD)	P value
**Trial allocation**	Intervention	488 (56%)	263 (40%)	<0.001
	Control	377 (44%)	398 (60%)	
**Site**	Liverpool	445 (51%)	397 (60%)	<0.001
	Cambridge	420 (49%)	264 (40%)	
**Age**	Up to 65 years	341 (39%)	270 (41%)	0.69
	66 – 70 years	379 (44%)	275 (42%)	
	Over 70 years	145 (17%)	116 (18%)	
**Gender**	Male	608 (70%)	463 (70%)	0.92
	Female	257 (30%)	198 (30%)	
**Marital group**	Married/cohabiting	593 (69%)	420 (64%)	0.05
	Not married/cohabiting	271 (31%)	239 (36%)	
**IMD**	Quintile 1 (most deprived)	269 (31%)	252 (38%)	<0.01
	Quintile 2	108 (12%)	80 (12%)	
	Quintile 3	133 (15%)	119 (18%)	
	Quintile 4	149 (17%)	92 (14%)	
	Quintile 5 (least deprived)	206 (24%)	118 (18%)	
**Lung cancer experience**	No	520 (60%)	342 (52%)	<0.001
	Yes	342 (40%)	316 (48%)	
**Cancer distress (T_0)⁺**		2.26 (0.28) *9.58*	2.24 (0.33) *9.39*	0.40

Note: percentages were calculated based on available data.

‡ Non-completers included non-responders and ineligible responders.

⁺ Log_n scores in normal text, original scores in italics (analyses performed using log_n scores).
