# Supplementary material for: Impact of low-dose CT screening on smoking cessation among high-risk participants in the UK Lung Cancer Screening Trial
Source: Thorax. 2017 Jul 14;72(10):912–8. doi: 10.1136/thoraxjnl-2016-209690 (PMC5738533; doi:10.1136/thoraxjnl-2016-209690)
Supplement: Supplementary table III [file thoraxjnl-2016-209690supp003.pdf]

Supplementary Table III. Predictors of T_1 smoking cessation using the imputed population

Quit smoking at T_1 (n=1544)					
		**Yes (n=111) n (%) or M (SD)**	**No (n=1433) n (%) or M (SD)**	**Univariable OR (95% CI)**	**Multivariable OR^ (95% CI)**
**Trial allocation**	Intervention	75 (68%)	683 (48%)	2.29 (1.52 to 3.45)	2.38 (1.56 to 3.64)
	Control	36 (32%)	750 (52%)		
**Site**	Liverpool	54 (49%)	801 (56%)	1.34 (0.91 to 1.97)	1.33 (0.79 to 2.25)
	Cambridge	57 (51%)	632 (44%)		
**Age group**	Up to 65 years	42 (38%)	572 (40%)	- Reference -	- Reference -
	66 – 70 years	52 (47%)	612 (43%)	1.16 (0.76 to 1.77)	1.18 (0.76 to 1.83)
	Over 70 years	17 (15%)	249 (17%)	0.93 (0.52 to 1.67)	0.98 (0.54 to 0.78)
**Gender**	Male	77 (69%)	1005 (70%)	1.04 (0.68 to 1.58)	1.01 (0.65 to 1.60)
	Female	34 (31%)	428 (30%)		
**Marital group**	Married/cohabiting	77 (70%)	943 (66%)	0.83 (0.54 to 1.26)	0.88 (0.56 to 1.39)
	Not married/cohabiting	33 (30%)	488 (34%)		
**IMD**	Quintile 1 (most deprived)	34 (31%)	495 (35%)	- Reference -	- Reference -
	Quintile 2	13 (12%)	177 (12%)	1.07 (0.55 to 2.07)	1.02 (0.50 to 2.04)
	Quintile 3	23 (21%)	230 (16%)	1.46 (0.84 to 2.53)	1.16 (0.61 to 2.19)
	Quintile 4	16 (14%)	228 (16%)	1.02 (0.55 to 1.89)	0.93 (0.46 to 1.89)
	Quintile 5 (least deprived)	25 (22%)	303 (21%)	1.20 (0.70 to 2.05)	1.00 (0.49 to 2.03)
**Lung cancer experience**	No	65 (60%)	808 (56%)	0.86 (0.57 to 1.28)	0.89 (0.59 to 1.36)
	Yes	43 (40%)	624 (44%)		
**Cancer distress (T_0)+**		2.30 (0.30) *9.97*	2.23 (0.30) *9.30*	2.02 (1.07 to 3.83)	2.43 (1.22 to 4.87)

^ Adjusted for T_0 cancer distress, recruitment site, gender, age, marital group, deprivation and experience of lung cancer.

+ Log_n scores in normal text, original scores in italics (analyses performed using log_n scores).
