# Supplementary material for: Impact of low-dose CT screening on smoking cessation among high-risk participants in the UK Lung Cancer Screening Trial
Source: Thorax. 2017 Jul 14;72(10):912–8. doi: 10.1136/thoraxjnl-2016-209690 (PMC5738533; doi:10.1136/thoraxjnl-2016-209690)
Supplement: Supplementary table IV [file thoraxjnl-2016-209690supp004.pdf]

**Supplementary Table IV. Predictors of $T_1$ smoking cessation using complete case analysis**

| Quit smoking at $T_1$ (n=1006) | | | | | |
|---|---|---|---|---|---|
| | | **Yes (n=111) n (%) or M (SD)** | **No (n=895) n (%) or M (SD)** | **Univariable OR (95% CI)** | **Multivariable OR^ (95% CI)** |
| **Trial allocation** | Intervention | 75 (68%) | 452 (50%) | 2.04 (1.34 to 3.10) | 2.09 (1.36 to 3.23) |
| | Control | 36 (32%) | 443 (50%) | | |
| **Site** | Liverpool | 54 (49%) | 486 (54%) | 1.26 (0.85 to 1.87) | 1.32 (0.76 to 2.32) |
| | Cambridge | 57 (51%) | 409 (46%) | | |
| **Age group** | Up to 65 years | 42 (38%) | 366 (41%) | - Reference - | |
| | 66 – 70 years | 52 (47%) | 392 (44%) | 1.16 (0.75 to 1.78) | 1.19 (0.76 to 1.86) |
| | Over 70 years | 17 (15%) | 137 (15%) | 1.08 (0.59 to 1.96) | 1.09 (0.59 to 2.01) |
| **Gender** | Male | 77 (69%) | 624 (70%) | 1.03 (0.67 to 1.59) | 0.99 (0.63 to 1.55) |
| | Female | 34 (31%) | 271 (30%) | | |
| **Marital group** | Married/cohabiting | 77 (70%) | 591 (66%) | 0.83 (0.54 to 1.27) | 0.86 (0.54 to 1.35) |
| | Not married/cohabiting | 33 (30%) | 302 (34%) | | |
| **IMD** | Quintile 1 (most deprived) | 34 (31%) | 312 (34%) | - Reference - | |
| | Quintile 2 | 13 (12%) | 96 (11%) | 1.25 (0.63 to 2.46) | 1.18 (0.58 to 2.41) |
| | Quintile 3 | 23 (21%) | 137 (15%) | 1.56 (0.89 to 2.76) | 1.21 (0.64 to 2.28) |
| | Quintile 4 | 16 (14%) | 146 (16%) | 1.02 (0.54 to 1.90) | 0.94 (0.46 to 1.92) |
| | Quintile 5 (least deprived) | 25 (22%) | 204 (23%) | 1.13 (0.65 to 1.95) | 0.95 (0.44 to 2.05) |
| **Lung cancer experience** | No | 65 (60%) | 535 (60%) | 0.97 (0.65 to 1.46) | 1.02 (0.67 to 1.58) |
| | Yes | 43 (40%) | 360 (40%) | | |
| **Cancer distress ($T_0$)$^+$** | | 2.30 (0.30) *9.97* | 2.23 (0.29) *9.30* | 2.15 (1.05 to 4.40) | 2.42 (1.14 to 5.12) |

^ Adjusted for $T_0$ cancer distress, recruitment site, gender, age, marital group, deprivation and experience of lung cancer.

$^+$ $Log_n$ scores in normal text, original scores in italics (analyses performed using $log_n$ scores).
