# Supplementary material for: Impact of low-dose CT screening on smoking cessation among high-risk participants in the UK Lung Cancer Screening Trial
Source: Thorax. 2017 Jul 14;72(10):912–8. doi: 10.1136/thoraxjnl-2016-209690 (PMC5738533; doi:10.1136/thoraxjnl-2016-209690)
Supplement: Supplementary table V [file thoraxjnl-2016-209690supp005.pdf]

Supplementary Table V. Predictors of T_2 smoking cessation using the imputed population

Quit smoking at T_2 (n=1524)					
		**Yes (n=194) n (%) or M (SD)**	**No (n=1330) n (%) or M (SD)**	**Univariable OR (95% CI)**	**Multivariable OR^ (95% CI)**
**Trial allocation**	Intervention	115 (59%)	634 (48%)	1.60 (1.18 to 2.17)	1.60 (1.17 to 2.18)
	Control	79 (41%)	696 (52%)		
**Site**	Liverpool	100 (51%)	741 (56%)	1.18 (0.87 to 1.60)	0.95 (0.64 to 1.41)
	Cambridge	94 (49%)	589 (44%)		
**Age (years)**	Up to 65 years	71 (37%)	540 (41%)	- Reference -	- Reference -
	66 to 70 years	84 (43%)	568 (43%)	1.11 (0.80 to 1.58)	1.11 (0.79 to 1.57)
	Over 70 years	39 (20%)	222 (17%)	1.34 (0.88 to 2.04)	1.36 (0.88 to 2.09)
**Gender**	Male	144 (74%)	925 (70%)	0.79 (0.56 to 1.12)	0.80 (0.56 to 1.15)
	Female	50 (26%)	405 (30%)		
**Marital group**	Married/cohabiting	140 (72%)	871 (66%)	0.74 (0.53 to 1.03)	0.80 (0.56 to 1.14)
	Not married/cohabiting	54 (28%)	456 (34%)		
**IMD**	Quintile 1 (most deprived)	55 (28%)	465 (35%)	- Reference -	- Reference -
	Quintile 2	25 (13%)	163 (12%)	1.30 (0.78 to 2.15)	1.26 (0.74 to 2.14)
	Quintile 3	34 (18%)	218 (16%)	1.32 (0.84 to 2.09)	1.15 (0.69 to 1.91)
	Quintile 4	37 (19%)	203 (15%)	1.54 (0.98 to 2.41)	1.53 (0.91 to 2.56)
	Quintile 5 (least deprived)	43 (22%)	281 (21%)	1.30 (0.85 to 1.98)	1.23 (0.71 to 2.14)
**Lung cancer experience**	No	117 (61%)	745 (56%)	0.81 (0.59 to 1.10)	0.85 (0.61 to 1.17)
	Yes	74 (39%)	584 (44%)		
**Cancer distress (T_0)⁺**		2.27 (0.28) *9.64*	2.23 (0.30) *9.33*	1.45 (0.88 to 2.39)	1.81 (1.07 to 3.08)

^ Adjusted for T_0 cancer distress, recruitment site, gender, age, marital group, deprivation and experience of lung cancer.

⁺ Log_n scores in normal text, original scores in italics (analyses performed using log_n scores).
