# Supplementary material for: Impact of low-dose CT screening on smoking cessation among high-risk participants in the UK Lung Cancer Screening Trial
Source: Thorax. 2017 Jul 14;72(10):912–8. doi: 10.1136/thoraxjnl-2016-209690 (PMC5738533; doi:10.1136/thoraxjnl-2016-209690)
Supplement: Supplementary table VI [file thoraxjnl-2016-209690supp006.pdf]

**Supplementary Table VI. Predictors of $T_2$ smoking cessation using complete case analysis**

| Quit smoking at $T_2$ (n=865) | | | | | |
|---|---|---|---|---|---|
| | | **Yes (n=194)** **n (%) or M (SD)** | **No (n=671)** **n (%) or M (SD)** | **Univariable OR (95% CI)** | **Multivariable OR^ (95% CI)** |
| **Trial allocation** | Intervention | 115 (59%) | 373 (55%) | 1.16 (0.84 to 1.61) | 1.16 (0.65 to 1.33) |
| | Control | 79 (41%) | 298 (45%) | | |
| **Site** | Liverpool | 100 (51%) | 350 (52%) | 1.03 (0.74 to 1.43) | 0.90 (0.59 to 1.37) |
| | Cambridge | 94 (49%) | 329 (48%) | | |
| **Age (years)** | Up to 65 years | 71 (37%) | 270 (40%) | - Reference - | - Reference - |
| | 66 to 70 years | 84 (43%) | 295 (44%) | 1.08 (0.75 to 1.55) | 1.08 (0.75 to 1.57) |
| | Over 70 years | 39 (20%) | 106 (16%) | 1.49 (0.94 to 2.37) | 1.54 (0.97 to 2.46) |
| **Gender** | Male | 144 (74%) | 471 (69%) | 0.76 (0.53 to 1.10) | 0.73 (0.50 to 1.07) |
| | Female | 50 (26%) | 208 (31%) | | |
| **Marital group** | Married/cohabiting | 140 (72%) | 458 (68%) | 0.86 (0.60 to 1.24) | 0.94 (0.64 to 1.38) |
| | Not married/cohabiting | 54 (28%) | 220 (32%) | | |
| **IMD** | Quintile 1 (most deprived) | 55 (28%) | 218 (32%) | - Reference - | - Reference - |
| | Quintile 2 | 25 (13%) | 83 (12%) | 1.21 (0.70 to 2.10) | 1.21 (0.69 to 2.13) |
| | Quintile 3 | 34 (18%) | 102 (15%) | 1.37 (0.83 to 2.25) | 1.25 (0.74 to 2.11) |
| | Quintile 4 | 37 (19%) | 112 (17%) | 1.38 (0.85 to 2.24) | 1.43 (0.82 to 2.49) |
| | Quintile 5 (least deprived) | 43 (22%) | 164 (24%) | 1.08 (0.68 to 1.71) | 1.12 (0.62 to 2.05) |
| **Lung cancer experience** | No | 117 (61%) | 408 (60%) | 0.91 (0.65 to 1.28) | 0.93 (0.65 to 1.33) |
| | Yes | 74 (39%) | 271 (40%) | | |
| **Cancer distress ($T_0$)$^+$** | | 2.27 (0.28) *10.01 (2.74)* | 2.23 (0.28) *9.63 (2.78)* | 1.69 (0.95 to 3.02) | 1.99 (1.08 to 3.67) |

^ Adjusted for $T_0$ cancer distress, recruitment site, gender, age, marital group, deprivation and experience of lung cancer.

$^+$ $Log_n$ scores in normal text, original scores in italics (analyses performed using $log_n$ scores).
